# PCB-95 Modulates the Calcium-Dependent Signaling Pathway Responsible for Activity-Dependent Dendritic Growth

**DOI:** 10.1289/ehp.1104833

**Published:** 2012-04-25

**Authors:** Gary A. Wayman, Diptiman D. Bose, Dongren Yang, Adam Lesiak, Donald Bruun, Soren Impey, Veronica Ledoux, Isaac N. Pessah, Pamela J. Lein

**Affiliations:** 1 Program in Neuroscience, Department of Veterinary and Comparative Anatomy, Pharmacology and Physiology, Washington State University, Pullman, Washington, USA; 2 Department of Molecular Biosciences, University of California, Davis, Davis, California, USA; 3 Oregon Stem Cell Center, and; 4 Center for Research on Occupational and Environmental Toxicology, Oregon Health & Science University, Portland, Oregon, USA

**Keywords:** autism, Ca^2+^, CaMKI, CREB, dendrites, developmental neurotoxicity, hippocampal MEK, neuronal connectivity, neurons, non-dioxin-like PCBs, ryanodine receptor, Wnt2

## Abstract

Background: Non-dioxin-like (NDL) polychlorinated biphenyls (PCBs) promote dendritic growth in hippocampal neurons via ryanodine receptor (RyR)-dependent mechanisms; however, downstream signaling events that link enhanced RyR activity to dendritic growth are unknown. Activity-dependent dendritic growth, which is a critical determinant of neuronal connectivity in the developing brain, is mediated by calcium ion (Ca^2+^)-dependent activation of Ca^2+^/calmodulin kinase-I (CaMKI), which triggers cAMP response element binding protein (CREB)-dependent Wnt2 transcription. RyRs regulate the spatiotemporal dynamics of intracellular Ca^2+^ signals, but whether RyRs promote dendritic growth via modulation of this signaling pathway is not known.

Objective: We tested the hypothesis that the CaMKI–CREB–Wnt2 signaling pathway couples NDL PCB-enhanced RyR activity to dendritic arborization.

Methods and Results: Ca^2+^ imaging of dissociated cultures of primary rat hippocampal neurons indicated that PCB-95 (2,2´,3,5´6-pentachlorobiphenyl; a potent RyR potentiator), enhanced synchronized Ca^2+^ oscillations in somata and dendrites that were blocked by ryanodine. As determined by Western blotting and quantitative polymerase chain reaction, PCB-95 also activated CREB and up-regulated Wnt2. Blocking CaMKK, CaMKIα/γ, MEK/ERK, CREB, or Wnt2 prevented PCB-95–induced dendritic growth. Antagonism of γ-aminobutyric acid (GABA) receptors with bicuculline (BIC) phenocopied the dendrite-promoting effects of PCB-95, and pharmacological antagonism or siRNA knockdown of RyR blocked BIC-induced dendritic growth in dissociated and slice cultures of hippocampal neurons.

Conclusions: RyR activity contributes to dynamic remodeling of dendritic architecture in response to NDL PCBs via CaMKI–CREB–Wnt2 signaling in rats. Our findings identify PCBs as candidate environmental risk factors for neurodevelopmental disorders, especially in children with heritable deficits in calcium signaling associated with autism.

Dendritic growth is strongly influenced by neuronal activity as evidenced by the remarkable effect of experience on the development and refinement of synaptic connections, which not only patterns neural circuitry during development but also underlies associative learning (Pittenger and Kandel 2003). The effects of neuronal activity on dendritic growth are mediated primarily, if not exclusively, by changes in intracellular calcium ion (Ca^2+^) levels (Lohmann and Wong 2005; Segal et al. 2000). We previously identified an NMDA (*N*-methyl-d-aspartate) receptor-mediated Ca^2+^-dependent signaling pathway that couples neuronal activity to dendritic arborization via sequential activation of Ca^2+^/calmodulin-dependent protein kinase kinase (CaMKK), Ca^2+^/calmodulin kinase-I (CaMKI), and extracellular signal-regulated kinase kinase (MEK/ERK) to enhance the cAMP response element binding protein (CREB)-mediated transcription of Wnt2 [wingless-type mouse mammary tumor virus (MMTV) integration site family member 2] (Wayman et al. 2006).

Polychlorinated biphenyls (PCBs) alter neuronal connectivity by interfering with normal patterns of dendritic growth and plasticity (Lein et al. 2007; Yang et al. 2009). Non-dioxin-like (NDL) PCBs promote dendritic growth via ryanodine receptor (RyR)-dependent mechanisms (Wayman et al. 2012); however, the downstream signaling events that mediate RyR-dependent effects of PCBs on dendritic arborization have yet to be described. In this study, we tested the hypothesis that the CaMKI–CREB–Wnt2 signaling pathway responsible for activity-dependent dendritic growth also couples NDL PCB-enhanced RyR activity to dendritic arborization. This hypothesis derives from observations that *a*) potentiation of RyR activity by NDL PCBs amplifies ionotropic glutamate receptor signaling (Gafni et al. 2004) and increases intracellular Ca^2+^ levels in neurons (Wong et al. 1997, 2001); *b*) Ca^2+^ signals generated by stimuli that promote dendritic growth, such as repetitive or prolonged depolarization (Emptage et al. 1999; Riquelme et al. 2011) or brain-derived neurotrophic factor (BDNF) (Adasme et al. 2011), are derived primarily from RyR-mediated Ca^2+^ release; and *c*) RyR-dependent Ca^2+^ signals activate CaM kinases, CREB, and transcription of genes encoding Ca^2+^-regulated proteins (Adasme et al. 2011; Dolmetsch et al. 1998; W Li et al. 1998). Our findings demonstrate that in cultured rat hippocampal neurons, RyR activity, triggered by either NDL PCBs or neuronal activity, contributes to dynamic remodeling of dendritic architecture via CaMKI–CREB–Wnt2 signaling.

## Methods

A complete listing of reagents is provided in Supplemental Material, p. 3 (http://dx.doi.org/10.1289/ehp.1104833). Animals were treated humanely and with regard for alleviation of suffering according to protocols approved by the Institutional Animal Care and Use Committees of Oregon Health & Science University, University of California, Davis, and Washington State University, Pullman.

*Cell culture.* Hippocampal neurons were dissociated from postnatal day–1 Sprague-Dawley rats (Charles River Laboratories, Wilmington, MA) and cultured at high density (10^5^ cells/cm^2^) in Neurobasal-A medium (Invitrogen, Carlsbad, CA) supplemented with B27 (Invitrogen) as described previously (Wayman et al. 2006). To visualize dendritic arbors, cultures were transfected at 6 days *in vitro* (DIV) with the plasmid-encoding microtubule-associated-protein-2B MAP2B (which labels the somatodendritic domain) fused to enhanced green fluorescent protein (EGFP) using Lipofectamine-2000 (Invitrogen) according to the manufacturer’s protocol. A subset of cultures was simultaneously transfected with plasmids encoding dominant negative (dn) CaMKI (dnCaMKI), dnCREB (also referred to as ACREB), or Wnt inhibitory factor (Wif). PCBs or vehicle (DMSO at 1:1000 dilution) was added to the culture medium for 48 hr beginning at 7 DIV; in a subset of cultures, a CaMK kinase inhibitor (STO-609, 5 μM) or a MEK inhibitor (U0126, 10 μM) was also added to the medium during the same period.

Organotypic hippocampal slices from postnatal day–5 rats were cultured for 3 days as described previously (Lein et al. 2011). At 3 DIV, slice cultures were biolistically transfected with plasmid-encoding tomato fluorescent protein (TFP) using the Helios gene gun (Bio-Rad, Hercules, CA) per the manufacturer’s directions. A subset of slice cultures was simultaneously transfected with siRNA (small interfering RNA) specific for RyR1 or RyR2. Slice cultures were exposed to vehicle, and PCBs were added to the culture medium during 4–6 DIV. A subset of cultures was also exposed to FLA365 [4-(2-aminopropyl)-3,5-dichloro-*N*,*N*-dimethylaniline] (10 μM), which was added to the culture medium during the same period.

Dendritic morphology was quantified from digital images of green fluorescent protein–positive (GFP^+^) or TFP^+^ neurons using Image J version 1.44p with the Neuron J plug-in version 1.42 to trace neurons (Meijering et al. 2004).

*Calcium imaging.* Spontaneous and electrically evoked Ca^2+^ transients were measured in dissociated hippocampal neurons cultured on Greiner CELLSTAR® micro-clear wells (Sigma-Aldrich, St. Louis, MO). Cells were loaded with the Ca^2+^-sensitive dye Fluo-4 AM (5 µM; Invitrogen) at 37°C for 30 min in imaging buffer consisting of 140 mM sodium choride (NaCl), 5 mM potassium chloride (KCl), 2 mM magnesium chloride (MgCl_2_), 2 mM calcium chloride (CaCl_2_), 10 mM HEPES, and 10 mM glucose, at pH 7.4, and supplemented with 0.05% BSA (bovine serum albumin). Cultures were washed three times with imaging buffer and transferred to the stage of an inverted Olympus IX70 microscope (Olympus America, Center Valley, PA) equipped with a 60 × 1.25 numeric aperture objective. Fluo-4 was excited at 494 nm using a DeltaRam illuminator (Photon Technologies Int’l., Birmingham, NJ); fluorescence emission was captured at 510 nm. Full-frame images were captured with an Evolve® cooled charge coupled device camera (Photometrics, Tucson, AZ) at 30 frames/sec (fps) using EasyRatioPro software (Photon Technologies Int’l.). In a subset of experiments, cultures were exposed to PCB-95 (2,2´,3,5´6-pentachlorobiphenyl; 2, 20, or 200 nM) from 7–9 DIV before loading with Fluo-4. After baseline recording, cultures were sequentially stimulated with electrical bipolar field pulses (0.5 millisec) at 1, 2.5, 5, and 10 Hz for 10 sec with 50-sec interstimulus rest periods using platinum electrodes connected to a Master 8 stimulator (A.M.P.I, Jerusalem, Israel). After acquisition, regions of interest were drawn freehand to encompass soma and distal dendrites (separated from the soma by a length of > 2 times the soma diameter). Movies were replayed to quantitatively measure changes in Fluo-4 fluorescence within the regions of interest.

At 7 DIV in separate experiments, spontaneous synchronized Ca^2+^ transients were measured from hippocampal neurons that had not been previously exposed to PCB-95 before loading with Fluo-A. PCB-95 (200 nM) or vehicle (DMSO at 1:1,000 dilution) in imaging buffer was acutely introduced into cultures by continuous flow perfusion (~ 1 mL/min). In a subset of cultures, ryanodine (500 µM; EMD Biosciences, Philadelphia, PA) was added to the culture medium 1 hr before loading the cells with Fluo-A to irreversibly block RyR channel activity (Buck et al. 1992; Zimanyi et al. 1992), which was verified by a challenge with 4-chloro-*m*-cresol (4CmC, 100 µM; Sigma-Aldrich). Changes in cytoplasmic Ca^2+^ were continuously recorded at 30 fps. Ca^2+^ transients > 2 times the baseline amplitude were scored as oscillations. The number of oscillations was compared between vehicle and PCB-95–treated neurons. Transient amplitude was measured by normalizing peak change in Fluo-A fluorescence (ΔF) to the fluorescence baseline (F_0_) and presented as mean ΔF/F_0_ for each neuron included in the analysis. Statistical comparisons were made using neurons (*n* = 30) obtained from three separate dissections. Statistical analysis was performed using unpaired Student’s *t* test.

*Quantitative polymerase chain reaction (qPCR).* Total RNA was isolated from dissociated hippocampal neuron cultures (9 DIV) using Trizol (Invitrogen) according to manufacturer’s instructions. Levels of *Wnt2* mRNA were quantified by qPCR and normalized to *GAPDH* (glyceraldehyde 3-phosphate dehydrogenase gene) mRNA levels in the same sample. Primer sequences and a more detailed description are provided in Supplemental Material, p. 4 (http://dx.doi.org/10.1289/ehp.1104833).

## Results

**Figure 1:**
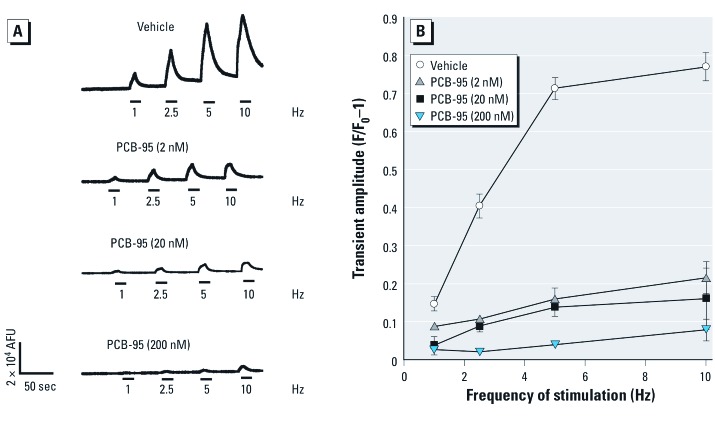
Prolonged PCB-95 exposure attenuates electrically evoked Ca^2+^ signals in cultured hippocampal neurons. (*A*) Representative Ca^2+^ transients produced by 9-DIV (days *in vitro*) neurons in response to electrical stimuli applied at increasing frequency (10-sec duration at 6 V). Neurons were loaded with Fluo-4 after 48-hr treatment with either vehicle or PCB-95. (*B*) Prolonged exposure to PCB-95 decreased the amplitude of electrically evoked Ca^2+^ transients in a concentration-dependent manner. AFU, arbitrary fluorescent units. Data are presented as mean ± SE (*n* = 10–12 neurons from three independent dissections). *p* < 0.05 relative to vehicle control for all treatment groups.

**Figure 2:**
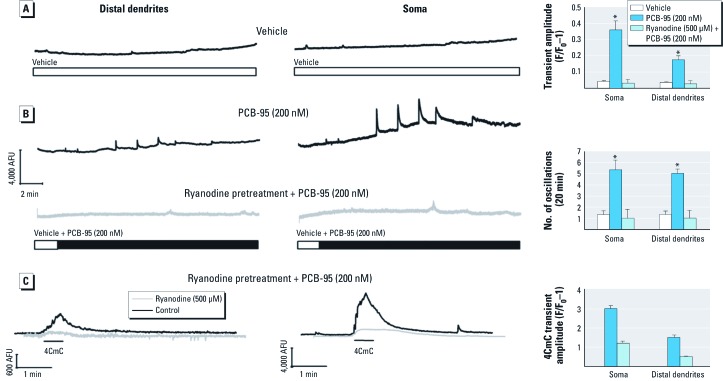
Ryanodine blocks amplification of spontaneous Ca^2+^ oscillations triggered by acute PCB-95 exposure. (*A*) Representative Ca^2+^ transient activity captured from soma and distal dendrites of Fluo-4–loaded hippocampal neurons at 7 DIV with perfusion of DMSO (0.01%) vehicle. (*B*) Representative Ca^2+^ transient activity captured from soma and distal dendrites of Fluo-4–loaded hippocampal neurons at 7 DIV before and after perfusion of PCB-95 (200 nM) (upper two traces). Neurons pretreated with ryanodine (500 µM, 1 hr) to block all RyR channel activity essentially negated the stimulatory actions of PCB-95 (lower two traces). (*C*) 4CmC (100 µM) which activates RyR1 and RyR2, but not RyR3, was used to confirm the block of Ca^2+^ channel activity. Bar graphs at the right summarize Ca^2+^ transient responses > 2× mean normalized to baseline fluorescence. Acute exposure to PCB-95 increased the frequency and amplitude of spontaneous Ca^2+^ transients in soma and distal dendrites. Data are presented as mean ± SE (*n* = 10–12 neurons from three independent dissections). *p < 0.05 relative to vehicle control.

*PCB-95 alters Ca^2+^ signals in cultured hippocampal neurons.* We used real-time Ca^2+^ imaging techniques to examine how subchronic (48 hr) or acute exposure to PCB-95 influences Ca^2+^ signaling behaviors in cultured hippocampal neurons. Exposure to PCB-95 during 7–9 DIV caused a concentration-dependent attenuation of the amplitude of electrically evoked Ca^2+^ transients (Figure 1A,B). This observation is consistent with previous findings that prolonged RyR activation by PCB-95 can deplete RyR-sensitive Ca^2+^ stores (Gafni et al. 2004; Morton-Jones et al. 2008). Evidence that the GABA_A_ (type A γ-aminobutyric acid) receptor antagonist bicuculline (BIC) triggers dendritic growth via transient rather than prolonged activation of Ca^2+^-dependent signaling pathways (Wayman et al. 2006) suggests that PCB-95 may similarly trigger dendritic growth via amplification of Ca^2+^ signals on a shorter time scale. We therefore examined Ca^2+^ oscillatory behavior in PCB-naïve hippocampal neurons loaded with the Ca^2+^ indicator Fluo-A at 7 DIV immediately before focal applications of vehicle or PCB-95. Although vehicle alone had no influence (Figure 2A), acute application of PCB-95 at 200 nM, a concentration that promotes robust dendritic growth, significantly enhanced the amplitude and oscillations of Ca^2+^ transients in both soma and distal dendrites of cultured rat hippocampal neurons (Figure 2B, top traces). Amplification of Ca^2+^ oscillations by PCB-95 was dependent on RyR activity as oscillations were completely prevented by pre-incubation of neurons with ryanodine (Figure 2B, bottom traces) under conditions previously shown to completely block gating of RyRs (500 µM, 1 hr) (Buck et al. 1992; Zimanyi et al. 1992). To verify that ryanodine pretreatment in fact blocked all RyR channel activity in hippocampal neurons, a challenge of 4CmC [previously shown to activate RyR1 and RyR2, but not RyR3, channel activity (Fessenden et al. 2003)], was performed at the end of each recording. Ryanodine pretreatment resulted in a near complete block of 4CmC responses (Figure 2C).

**Figure 3:**
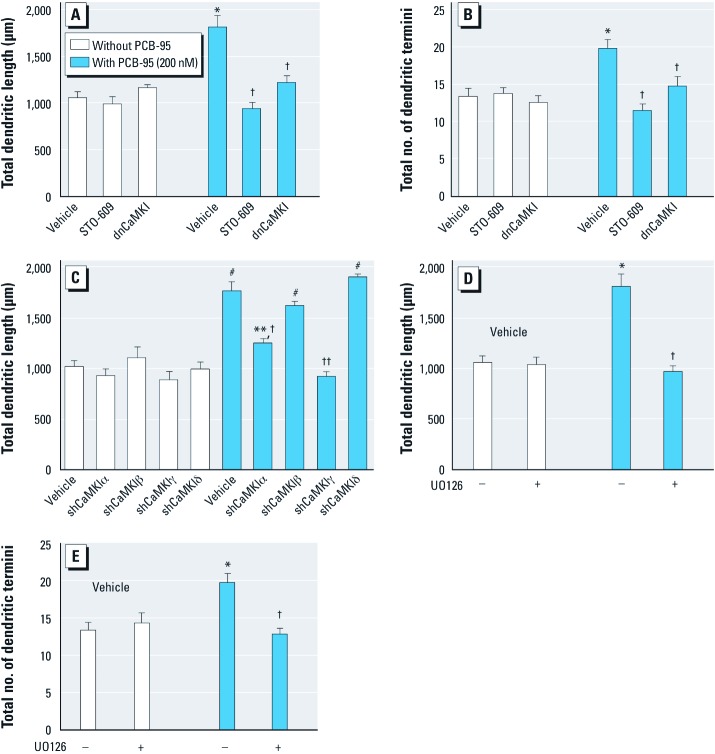
PCB-95–induced dendritic growth requires CaMKK/CaMKI and MEK signaling. Dendritic length (*A*) and branching (*B*) were quantified in MAP2B-EGFP^+^ hippocampal neurons (9 DIV) treated with vehicle or PCB-95 for 48 hr after pharmacological blockade of CaMKI by STO-609 (5 µM) or expression of dnCaMKI. (*C*) Transfection with shRNA for CaMKIα or CaMKIγ, but not CaMKIβ or CaMKIδ, blocked PCB-95–induced dendritic growth. Inhibiting downstream MEK signaling with 10 µM U0126 also blocked PCB-95 effects on dendritic length (*D*) and branching (*E*) (–, without, +, with U0126). Data are presented as mean ± SE (*n *= 30 neurons). *p < 0.05, **p < 0.01, and ^#^*p* < 0.001 relative to vehicle control. ^†^*p* < 0.05, and ^††^*p* < 0.01 relative to PCB-95 alone.

*CaMKI–CREB–Wnt2 signaling mediates PCB-95–induced dendritic growth*. Previous reports demonstrated that treatment of cultured hippocampal neurons with the CaMK kinase inhibitor STO-609 (2–5 μM) inhibits CaMKI but not CaMKII (Wayman et al. 2004). STO-609 (5 μM) selectively blocked PCB-95 effects on dendritic length (Figure 3A) and branching (Figure 3B) without altering basal dendritic growth. Expression of a previously characterized dnCaMKI (Wayman et al. 2004), also suppressed the stimulatory effects of PCB-95 on dendritic length and branching with no effect on basal parameters (Figure 3A,B). To determine which CaMKI isoforms may be involved in PCB-95–induced dendritic growth, previously characterized small hairpin RNA (shRNA) constructs that suppress individual CaMKI isoforms (Wayman et al. 2006) were expressed in hippocampal neurons. Expression of CaMKIα shRNA significantly reduced and CaMKIγ shRNA blocked the stimulatory effects of PCB-95 on dendrites; in contrast, shRNAs for β and δ CaMKI had no effect (Figure 3C).

To probe downstream effectors of CaMKI, we first determined whether PCB-95–induced dendritic growth requires MEK/ERK signaling. Western blot analyses of phosphorylated ERK (pERK) levels did not consistently detect significantly increased pERK at 5–30 min after acute exposure of 7-DIV hippocampal cultures to PCB-95 (data not shown). However, the MEK inhibitor U0126 (10 μM) completely blocked the stimulatory effects of PCB-95 on dendritic length and branching in the absence of any effect on basal dendritic growth (Figure 3D,E).

**Figure 4:**
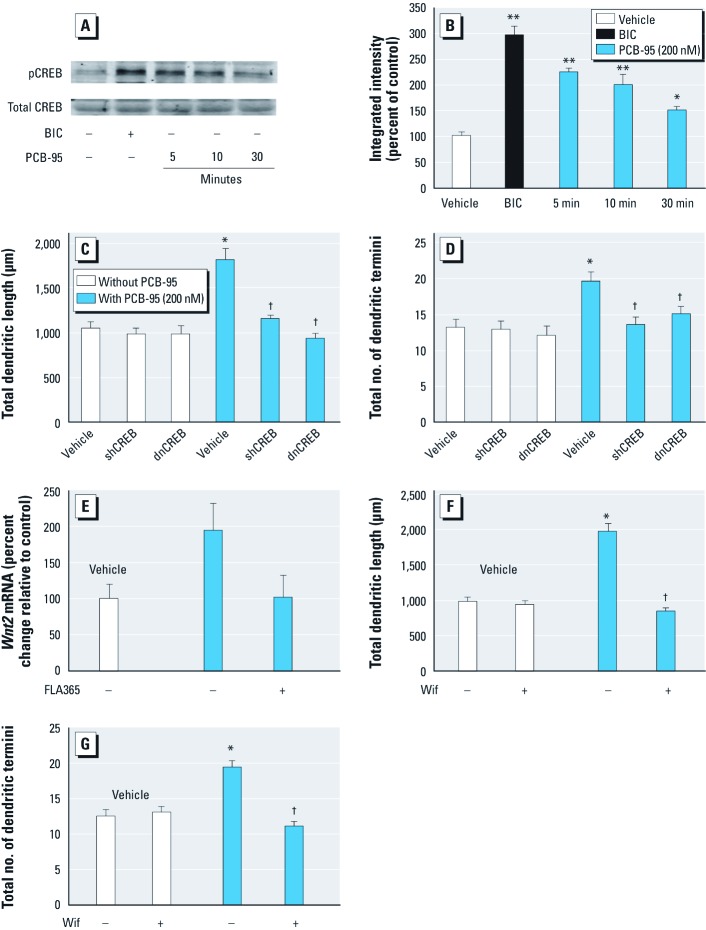
CREB and Wnt2 activity are required for PCB-95–induced dendritic growth. (*A*) Representative Western blot illustrating increased pCREB in hippocampal neurons (7 DIV) stimulated with BIC (20 µM, 30 min) or PCB-95 (200 nM). (*B*) Densitometry of blots probed for pCREB normalized to total CREB presented as percent of control (mean ± SE; *n *= 3). Expression of dnCREB or CREB shRNA inhibited PCB-95 effects on dendritic length (*C*) and branching (*D*) in 9-DIV hippocampal neurons co-transfected with MAP2B-EGFP (mean ± SE; *n *= 30 neurons). (*E*) PCB-95 increased *Wnt2* mRNA in hippocampal neurons as determined by qPCR, and this effect was blocked by 10-µM FLA365 (mean ± SE; *n *= 3 per condition). Transfection with the endogenous Wnt2 antagonist Wif blocked PCB-95 effects on dendritic length (–, without, +, with FLA365) (*F*) and branching (*G*) in 9-DIV hippocampal neurons co-transfected with MAP2B-EGFP (–, without, +, with Wif) (mean ± SE; *n *= 30 neurons). *p < 0.05, and **p < 0.01 relative to vehicle control. ^†^*p* < 0.05 relative to PCB-95 alone.

Western blot analyses revealed that, as previously reported (Wayman et al. 2006), BIC increased phosphorylation of the active site (Ser133) of CREB (Figure 4A,B). PCB-95 similarly elicited rapid (< 5 min) and sustained (> 30 min) CREB phosphorylation. Expression of either dnCREB or CREB-specific shRNA blocked the effects of PCB-95 on dendritic length and branching but had no effect on dendritic growth in vehicle control cultures (Figure 4C,D). We previously showed that expression of either dnCREB or shCREB (short hairpin CREB) does not induce nuclear pyknosis, increase levels of cleaved caspase-3, or significantly alter MAP2B-EGFP expression or dendritic morphology in hippocampal neurons grown in the presence of the serum-free supplement, B27 (Wayman et al. 2006).

Activity-dependent CREB activation stimulates dendritic growth in part via increased Wnt2 expression (Wayman et al. 2006). Stimulation of hippocampal neurons with PCB-95 triggered a doubling of *Wnt2* mRNA and this effect was blocked by treatment with the selective RyR antagonist FLA365 (Figure 4E). Expression of the Wnt inhibitor Wif, a secreted protein that binds Wnt and prevents it from activating its receptor Frizzled (Han and Lin 2005), completely suppressed PCB-induced dendritic growth without altering basal dendritic growth (Figure 4F,G).

**Figure 5:**
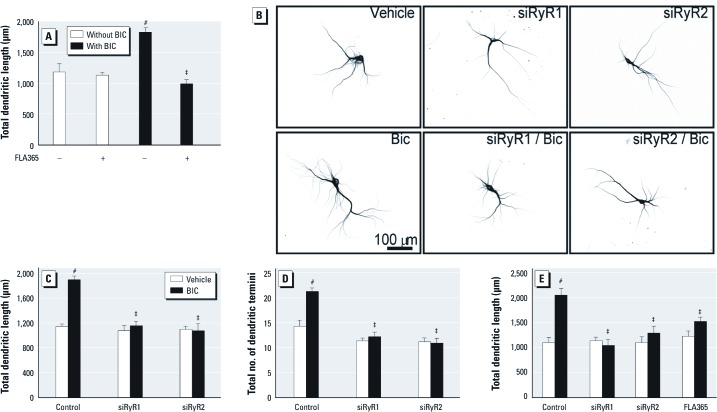
RyR activity is required for activity-induced dendritic growth. (*A*) A 48-hr exposure to BIC (20 μM) significantly increased dendritic growth in 9-DIV cultures of dissociated hippocampal neurons, and this effect was blocked by 10-µM FLA365 (–, without, +, with FLA365). (*B*) Representative photomicrographs of 9-DIV hippocampal neurons transfected with MAP2B-EGFP alone or in combination with RyR isoform-specific siRNA before 48-hr treatment with vehicle or BIC. Transfection with siRNA for RyR1 or RyR2 blocked BIC effects on dendritic length (*C*) and branching (*D*) in GFP^+^ hippocampal neurons grown in dissociated cultures. (*E*) BIC-induced dendritic growth in hippocampal slice cultures was blocked by pharmacological antagonism or siRNA knockdown of RyR. Data are presented as mean ± SE (*n *= 30 neurons). ^#^*p* < 0.001 relative to vehicle control. ^‡^*p* < 0.001 relative to PCB-95 alone.

*BIC-induced dendritic growth requires RyR activity*. These data raised the question of whether RyR activity is required for activity-dependent dendritic growth in the absence of PCBs. To address this question we used our previously described model of activity-dependent dendritic growth in which the GABA_A_ receptor antagonist BIC promotes dendritic growth in cultured hippocampal neurons via activation of NMDA receptors (Wayman et al. 2006). Consistent with our previous studies, BIC (20 μM) enhanced dendritic growth in cultures of dissociated hippocampal neurons (Figure 5A). BIC-induced dendritic growth was completely blocked by the RyR channel blocker FLA365 (Figure 5A). Expression of siRNA specific for either RyR1 or RyR2 [for characterization of siRNA specificity, see Supplemental Material, p. 4 and Figure S1 (http://dx.doi.org/10.1289/ehp.1104833)] completely suppressed BIC stimulation of dendritic length and branching but had no effect on basal dendritic growth (Figure 5B–D). Similarly, BIC enhanced dendritic arborization of pyramidal neurons in hippocampal slice cultures, and this effect was blocked by FLA365 and by siRNA knockdown of RyR1 or RyR2 (Figure 5E).

## Discussion

The major finding of this study is that in cultured rat hippocampal neurons, RyR activity, triggered by either NDL PCBs or neuronal activity, contributes to dynamic remodeling of dendritic architecture via Ca^2+^-dependent activation of the CaMKI–CREB–Wnt2 signaling pathway. We previously demonstrated that activity-dependent dendritic growth in hippocampal neurons is mediated by sequential activation of NMDA receptors, CaMKK, CaMKI, Ras, MEK/ERK, and CREB-dependent transcription of Wnt2 (Wayman et al. 2006). In this study, we confirmed that PCB-95 increases spontaneous Ca^2+^ oscillations in hippocampal neurons in a manner blocked by ryanodine, and we identified these same signaling molecules as downstream effectors that couple RyR activation to dendritic growth. Previous studies in cultured neurons demonstrated that the commercial PCB mixture Aroclor 1254 (A1254) (Inglefield et al. 2001) and RyR activity (Kemmerling et al. 2007) increased neuronal levels of pCREB. We confirmed that PCB-95 activated CREB in hippocampal neurons as determined by Western blot analyses of pCREB expression, and we extended this finding to demonstrate that PCB-95 also up-regulated expression of the CREB-responsive gene *Wnt2*, as determined by qPCR. More importantly, we established a crucial role for not only CREB and Wnt2, but also CaMKK–CaMKI and MEK/ERK signaling in RyR-dependent dendritic growth using multiple experimental approaches including pharmacological inhibition and expression of dominant-interfering or si/shRNA constructs specific for each of these signaling molecules. Consistent with our earlier studies of activity-dependent dendritic growth in cultured hippocampal neurons (Wayman et al. 2006), shRNAs against both α and γ CaMKI blocked the stimulatory effects of PCB-95 on dendrites, whereas shRNAs for β and δ CaMKI had no effect.

**Figure 6:**
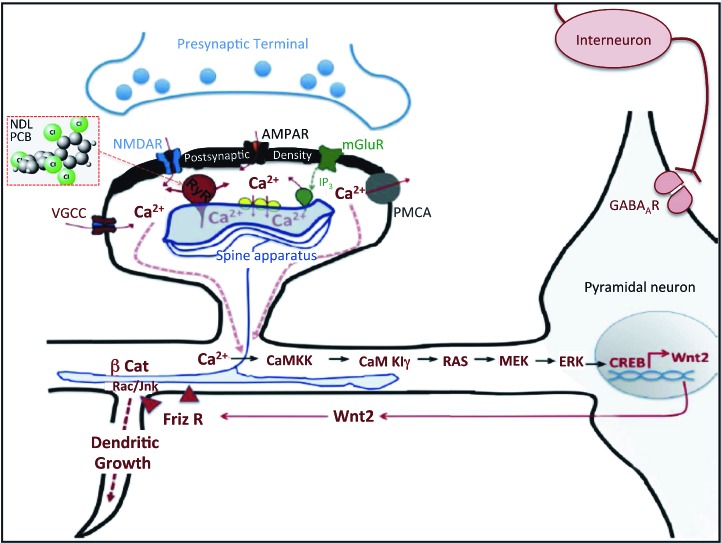
Schematic representation of RyR-dependent signaling pathway that mediates PCB-induced and activity-dependent dendritic growth. In the context of activity-dependent dendritic growth, RyR1 and RyR2 in the endoplasmic reticulum of the dendrite functionally link Ca^2+^ influx via NMDA and AMPA receptors to Ca^2+^ release from intracellular stores. Increased intracellular Ca^2+^ as a consequence of RyR activation sequentially activates the CaMKK–CaMK–Ras–MEK/ERK–CREB–Wnt2 signaling pathway to promote dendritic growth. Abbreviations: AMPA, a-amino-3-hydroxy-5-methyl-4-isoxazolepropionic acid; βCat, β-catenin; Friz, frizzled, the cognate receptor for Wnt2; Ip_3_, inositol 1,4,5-triphosphate; mGlu, metabotropic glutamate; PMCA, plasma membrane calcium ATPase; R, receptor; VGCC, voltage-gated Ca^2+^ channel.

These data suggest a model in which PCB-95 potentiation of RyR activity promotes dendritic growth by triggering the same Ca^2+^-dependent signaling pathway linked to activity-dependent dendritic growth in hippocampal neurons (Wayman et al. 2006). We propose that PCB-95 interacts with RyR channels to increase the amplitude and frequency of intracellular Ca^2+^ oscillations, which then trigger sequential activation of CaMKK, CaMKI, and MEK/ERK, resulting in the CREB-activated transcription of Wnt2 (Figure 6). Wnt2, a secreted glycoprotein, binds to the Frizzled family of receptors to activate the scaffold protein Dishevelled, which in turn stimulates dendritic growth via effects on the actin and microtubule cytoskeleton mediated by β-catenin and/or activation of the RhoGTPases Rac and/or JNK [members of the rho family of guanosine triphosphate (GTP) hydrolases] (Rosso et al. 2005; Yu and Malenka 2003). In our model, PCB-95 triggers this signaling pathway downstream of the NMDA receptor. However, RyRs are functionally coupled to NMDA receptors (Berridge 2006; Pessah et al. 2010), which considered in the context of our previous studies demonstrating that inhibition of NMDA receptors blocks activity-dependent dendritic growth (Wayman et al. 2006), suggested that RyR activity is required for not only PCB-induced dendritic growth, but also activity-dependent dendritic growth. In support of this hypothesis, we observed that the selective RyR antagonist, FLA365, as well as siRNA knockdown of either RyR1 or RyR2, completely blocked BIC-induced dendritic growth in both dissociated cultures of hippocampal neurons and hippocampal slice cultures.

Our observations add to the emerging experimental evidence supporting a central role for RyRs in neuronal excitability and use-dependent synaptic plasticity (Adasme et al. 2011; Bardo et al. 2006; Berridge 2006). Functional aspects of neuroplasticity, including long-term potentiation (Lu and Hawkins 2002; Wang et al. 1996), long-term depression (ST Li et al. 1998; Wang et al. 1997) and associative memory (Alkon et al. 1998; Baker et al. 2010; Edwards and Rickard 2006; Galeotti et al. 2008) are altered by experimental manipulation of RyR activity by ligands such as the RyR accessory FK506-binding protein (FKBP12) that directly modulate RyR or by agents that deregulate RyR by disrupting the RyR/FKBP12 complex. Spatial learning is tightly correlated with selective up-regulation of RyR expression (Adasme et al. 2011; Cavallaro et al. 1997; Yang et al. 2009; Zhao et al. 2000). Targeted deletion of RyR3 in mice causes impairments in social behavior (Matsuo et al. 2009) and deficits in contextual fear conditioning but improves spatial learning in the Morris water maze task (Futatsugi et al. 1999; Kouzu et al. 2000), whereas selective knockdown of RyR2 and RyR3 impairs avoidance memory processes (Galeotti et al. 2008). Previous studies have implicated a local release of Ca^2+^ from intracellular stores in the maintenance of dendrites (Lohmann et al. 2002), the regulation of the motility of dendritic filopodia (Lohmann et al. 2005), and the increased size of dendritic spines (Korkotian and Segal 1999). More recently, inhibitory concentrations of ryanodine were reported to block BDNF-enhanced spine formation in primary hippocampal neurons (Adasme et al. 2011). However, our findings are the first to demonstrate that RyR activity is sufficient to promote dendritic growth and is necessary for activity-dependent dendritic growth.

Defective neuronal connectivity is a common pathological feature in most neurodevelopmental disorders, including autism spectrum disorders (ASD) (Bourgeron 2009; Geschwind and Levitt 2007; Rubenstein and Merzenich 2003), which suggests that NDL PCBs are environmental risk factors for ASD. There is experimental evidence that developmental exposure to PCB-95 elicits some aspects of ASD, including an imbalance between excitation and inhibition in the auditory cortex of weanling rats (Kenet et al. 2007) and altered social behaviors in rats (Jolous-Jamshidi et al. 2010). However, it seems more likely that NDL PCBs interact with ASD-linked genes that converge on common signaling pathways to increase ASD risk and/or severity. Emerging evidence suggests that defective neuronal connectivity associated with ASD is mediated in part by defects in neuronal Ca^2+^ signaling (Lohmann 2009). A number of genes that encode proteins that have a primary role of generating intracellular Ca^2+^ signals or are themselves tightly regulated by local fluctuations in Ca^2+^ concentrations, including CREB and Wnt2, have been linked to ASD (Krey and Dolmetsch 2007; Levitt and Campbell 2009; Pessah and Lein 2008). More compelling are recent studies of a gain-of-function missense mutation in the L-type Ca^2+^ channel Ca_V_1.2 that causes Timothy syndrome, which has a 60% rate of co-morbidity with autism (Splawski et al. 2004), making it one of the most penetrant monogenic forms of ASD. Neurons differentiated from induced pluripotent stem cells derived from patients with Timothy syndrome revealed increased Ca^2+^ oscillations and up-regulated expression of genes linked to Ca^2+^-dependent regulation of CREB, including CaMK (Pasca et al. 2011). Such studies clearly establish these Ca^2+^-signaling molecules as possible convergence points for genetic variants linked to ASD risk and environmental factors such as NDL PCBs.

## Supplemental Material

(213 KB) PDF
