# Supplementary material for: PCB-95 Modulates the Calcium-Dependent Signaling Pathway Responsible for Activity-Dependent Dendritic Growth
Source: Environ Health Perspect. 2012 Apr 25;120(7):1003–9. doi: 10.1289/ehp.1104833 (PMC3404671; doi:10.1289/ehp.1104833)
Supplement: (213 KB) PDF [file ehp.1104833.s001.pdf]

Supplemental Material

PCB 95 Modulates Calcium-Dependent Signaling Pathway Responsible for Activity-Dependent Dendritic Growth

Gary A. Wayman[1*], Diptiman D. Bose[2*], Dongren Yang[2], Adam Lesiak[1], Donald Bruun[2], Soren Impey[3], Veronica Ledoux[4], Isaac N. Pessah[2] and Pamela J. Lein[2, 4]†

[1]Program in Neuroscience, Department of Veterinary and Comparative Anatomy, Pharmacology and Physiology, Washington State University, Pullman, WA 99164; [2]Department of Molecular Biosciences, University of California, Davis, CA 95616; [3]Oregon Stem Cell Center and [4]Center for Research on Occupational and Environmental Toxicology, Oregon Health & Science University, Portland, OR 97239

***These authors contributed equally to this work**

†Correspondence should be addressed to:

Pamela J. Lein, PhD

UC Davis School of Veterinary Medicine, Department of Molecular Biosciences

One Shields Avenue, 1120 Haring Hall, Davis, CA 95616

Telephone: (530) 752-1970; Fax: (530) 752-4698; Email: pjlein@ucdavis.edu

Table of Contents

	Page
Materials	3
Table 1. Sequences of RyR siRNA used in neuronal cells	4
WNT2 quantitative polymerase chain reaction (qPCR)	4
HEK cell culture and transfection	4
Supplemental References	5
Figure 1. Specificity of RyR siRNAs	6

Supplemental Methods

Materials

PCB 95 (2,2′,3,5′,6-pentachlorobiphenyl, >99% purity) and PCB 66 (2,3',4,4'-tetrachloro-biphenyl, >99% purity) were purchased from AccuStandard, Inc. (New Haven, CT). FLA365, which blocks RyR channels (Mack et al. 1992) was a generous gift from Michele Chiesi (CIBA). Bicuculline was purchased from Sigma (St. Louis, MO); U0126, from Calbiochem (EMD4Biosciences, Rockland, MA); and STO-609, from Tocris Biosciences (Ellisville, Missouri). Construction and characterization of the following expression vectors were previously published (Wayman et al. 2006): Microtubule-associated protein 2B (MAP2B)-enhanced green fluorescent protein (EGFP) fusion construct subcloned into the pCAGGS expression vector, pCAG-tomato fluorescent protein (TFP) and Wif expression vectors. Also previously described and characterized: plasmids encoding dominant negative (dn)CaMKI (K49E, T177A, IHQS286DDEE, F307A) (Wayman et al. 2004) and pCAGACREB (Arthur et al. 2004), and small hairpin (sh)RNA constructs targeting the α, β, γ and δ isoforms of Ca^{2+}/calmodulin kinase (CaMK)I subcloned into the p702P vector (Wayman et al. 2006). A detailed description and characterization of RyR-specific and negative control siRNA primers (Invitrogen, Carlsbad, CA) is provided in the supplemental material for the companion paper (Wayman et al. 2012). Three different sets of siRNA primers with differing GC levels were designed for RyR1 and for RyR2 using the Invitrogen (Carlsbad, CA) stealth RNAi service. Each set (which was a mixture of 2 separate siRNAs) was tested for efficiency and specificity using HEK cells stably transfected with either *Ryr1* or *Ryr2*. Data from these experiments was used to select one set against each RyR isoform to use in experiments in neuronal cell cultures (described in Table S1). The Invitrogen negative control kit were used as controls; sequences of these negative control siRNA are proprietary but are confirmed by Invitrogen to not be homologous to any expressed genes in the vertebrate transcriptome.

Supplemental Material, Table 1. Sequences of RyR siRNA used in neuronal cells

RyR1-specific siRNA	RyR2-specific siRNA
CGAGGAACUUCUACACGCUGCGAUU<br>and<br>AAUCGCAGCGUGUAGAAGUUCCUCG	CAAGCGCAUCGAGAGGGUCUACUUU<br>and<br>AAAGUAGACCCUCUCGAUGCGCUUG

WNT2 quantitative polymerase chain reaction (qPCR)

Total RNA was isolated from dissociated hippocampal neuron cultures (9 DIV) using Trizol (Invitrogen) according to manufacturer's instructions. RNA (50 ng to 3 mg) was reverse transcribed with Moloney murine leukemia virus reverse transcriptase (Invitrogen) and 50–250 ng random primers (Invitrogen). qPCR primers were designed by MIT's Primer3 software (http://frodo.wi.mit.edu/cgi-bin/primer3/primer3_www.cgi) using default parameters with the following exceptions: rodent and simple repeat library was on, product size was 50–200bp, primer size was 18–27 bases, Tm was 66ºC–72ºC, maximum self-complementarity was 5, and maximum 30 complementarity was 3. Primer sequences for Wnt2 are: forward GGC GTT GTA TTT GCC ATC ACC AG; reverse GCT GTC CTT GCC ACT CCC TTT C. PCRs (10ml) contained 1 ml 103 PCR buffer (Invitrogen), 2.5 mM MgCl2, 200 mM dNTP (Roche), 0.125–0.25 mM primer (IDT), 13 SYBR green I (Invitrogen), and 1 U platinum Taq (Invitrogen). PCR was run on an Opticon OP346 (MJ Research) for one cycle at 95ºC for 35 s, and 30–50 cycles at 94ºC for 15 s; 68ºC–70ºC for 40 s. RT-PCR experiments were normalized to GAPDH RNA levels (other housekeeping genes showed similar results). All RT-PCR generated 100-fold higher levels of product relative to no reverse transcriptase controls.

HEK cell culture and transfection

Human embryonic kidney (HEK 293) cells were maintained in DMEM medium supplemented with 2 mM glutamine, 100 μg/ml streptomycin, 100 U/ml penicillin, 1 mM sodium pyruvate, and 10% FBS. Cells were transfected with full-length cDNA for rabbit *Ryr1* or *Ryr2* cloned into the

pCI-neo expression vector (Promega, Madison, WI) using Lipofectamine 2000 (Invitrogen) according to the manufacturer's instructions. Stably transfected cells were obtained by Geneticin sulfate (500 μg/ml) selection for 2 wk. RyR1 and RyR2 expression were confirmed by immunocytochemistry using antibody 34C (University of Iowa Hybridoma Bank, Iowa City, IA) or C3-33 (generous gift of Dr. G. Meissner, University of North Carolina), respectively. Cultures were transfected with siRNA (25 pmol) using Lipofectamine RNAiMax (Invitrogen) per the manufacturer's instructions. RyR expression was quantified by in-cell western 48 h after transfection using anti-RyR1 antibody 34C (1:25) or anti-RyR2 antibody C3-33 (1:1000), and counterstaining with the nucleic acid binding dye DraQ5 (LICOR-Biosciences). Cultures were scanned using the LICOR Odyssey infrared imager. Total RyR signal (800 nm) was normalized to DraQ5 signal (700 nm) within the same well. Three wells were analyzed per treatment; significant differences were identified using one-way ANOVA with *post hoc* Newman-Keuls.

Supplemental References

Arthur JS, Fong AL, Dwyer JM, Davare M, Reese E, Obrietan K, et al. 2004. Mitogen- and stress-activated protein kinase 1 mediates cAMP response element-binding protein phosphorylation and activation by neurotrophins. J Neurosci 24(18):4324-4332.

Mack WM, Zimanyi I, Pessah IN. 1992. Discrimination of multiple binding sites for antagonists of the calcium release channel complex of skeletal and cardiac sarcoplasmic reticulum. The Journal of pharmacology and experimental therapeutics 262(3):1028-1037.

Wayman GA, Impey S, Marks D, Saneyoshi T, Grant WF, Derkach V, et al. 2006. Activity-dependent dendritic arborization mediated by CaM-kinase I activation and enhanced CREB-dependent transcription of Wnt-2. Neuron 50(6):897-909.

Wayman GA, Yang D, Bose DD, Lesiak A, Ledoux V, Bruun D, et al. 2012. PCB 95 promotes dendritic growth via ryanodine receptor-dependent mechanisms. Environ Health Perspect [Online xApril, 2012].

Wayman GA, Kaech S, Grant WF, Davare M, Impey S, Tokumitsu H, et al. 2004. Regulation of axonal extension and growth cone motility by calmodulin-dependent protein kinase I. J Neurosci 24(15):3786-3794.

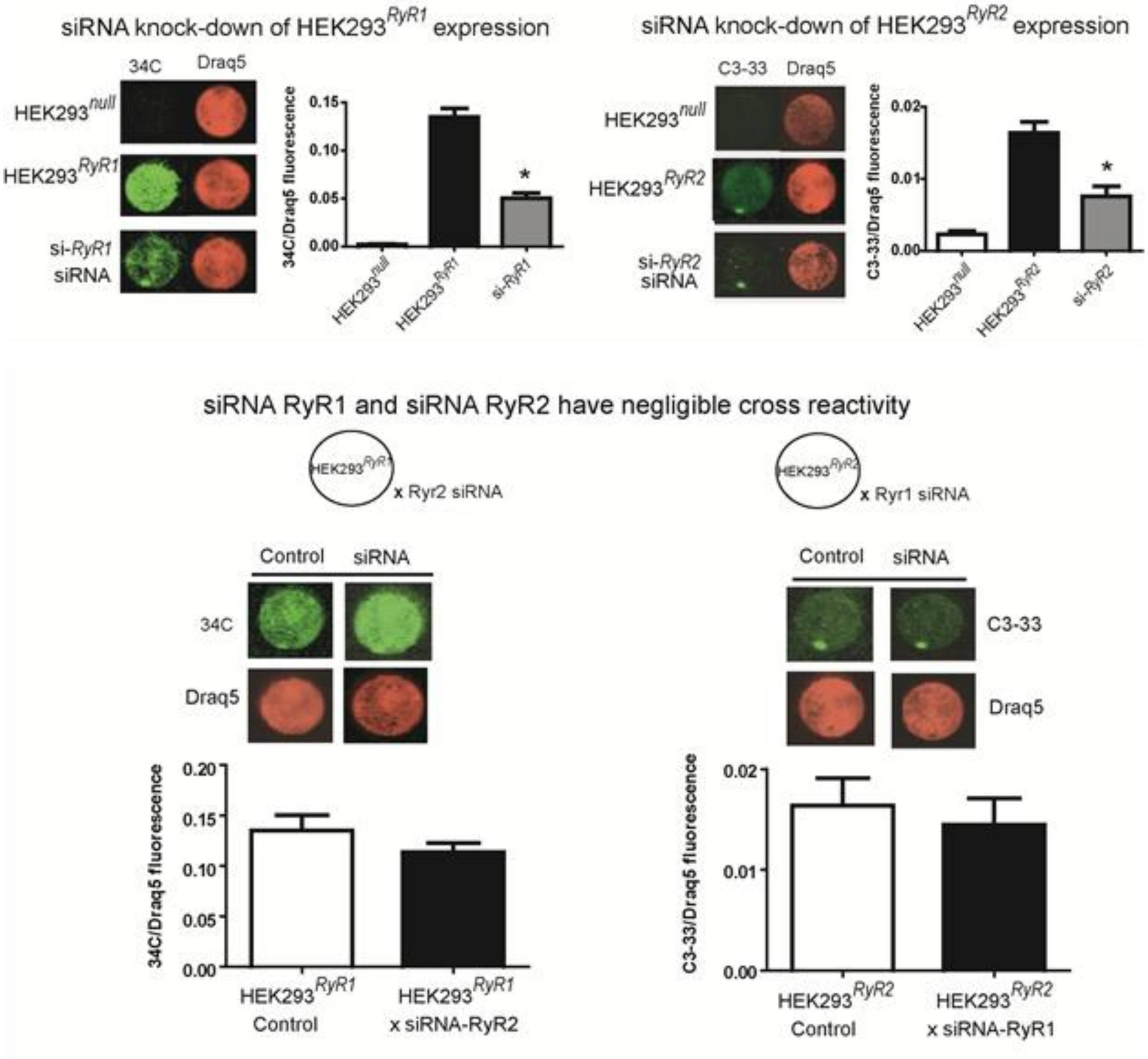

Supplemental Material, Figure 1. Specificity of RyR siRNAs. Wild type HEK293 cell line (HEK293^{null}), which lack detectable RyR expression, were stably transfected with *Ryr1* cDNA (HEK293^{RyR1}, top left) or *Ryr2* cDNA (HEK293^{RyR2}, top right).. RyR expression was quantified via densitometry of in-cell western blots 48 h after transfection with RyR isoform-specific siRNA constructs. Shown are representative images of in-cell western blots using antibodies 34C and C3-33 to probe RyR1 and RyR2, respectively, and Draq5 to stain nuclei; densitometric analyses of RyR1 and RyR2 expression normalized to Draq5 within each well are summarized in the bar graphs. As illustrated in bottom panels, siRNA 2-RyR1 did not knockdown RyR2 expression in HEK293^{RyR2} cells; conversely, siRNA 1-RyR2 did not knockdown RyR1 expression in HEK293^{RyR1} cells. Data expressed as the mean ± SD (n=3 wells).
